# Implications of proteome allocation constraints for understanding interbacterial antagonism

**DOI:** 10.1099/mic.0.001764

**Published:** 2026-09-04

**Authors:** Sean C. Booth

**Affiliations:** 1 Department of Microbiology, University of Manitoba, Winnipeg, MB, Canada

**Keywords:** antagonism, competition, proteome

## Abstract

Bacteria live in dense communities where competition influences the composition and, therefore, the function of these communities. Beyond competing for resources, bacteria engage in antagonism by deploying a range of molecular weapon systems to inhibit and kill other bacteria. Investing in antagonism is expected to incur a fitness trade-off, but the nature of this trade-off at the level of molecular physiology remains underexplained. Applying recent advances about the physiological constraints faced by bacterial cells may help us better understand existing studies and design new investigations into interbacterial antagonism. Bacterial cells face two important constraints: a finite amount of protein and a maximum translation speed for ribosomes. As a result, the only way for a cell to grow faster is to allocate more of its finite proteome to synthesizing ribosomes. A cell choosing to attack competitors must therefore allocate some of its limited proteome budget to antagonistic proteins instead of other functions. Conversely, being attacked and resisting the effects of such attacks also require an investment of proteomic resources. The extent to which proteome allocation constraints influence bacterial physiology is not fully understood; consequently, how these constraints influence interbacterial antagonism has not been investigated. Here, I will discuss how proteome allocation constraints can re-contextualize our existing understanding of the costs of both deploying and resisting attacks and how investigation of these constraints may further our understanding of interbacterial antagonism.

## Interbacterial antagonism influences the composition of important microbial communities

Across a wide range of environments, bacteria live in complex communities composed of diverse members from all taxonomic levels [1]. While more distantly related species may coexist through niche partitioning and even cooperate with each other [2], closely related strains must compete for the same resources [3]. Competition is indeed expected to be the most prevalent interaction within bacterial communities [4]. These competitive interactions are thus important for determining the presence and abundance of community members, which, in turn, determine the community’s function [1]. Bacterial community function can impact human well-being at the level of individual people: the gut microbiome influences resistance to pathogens [5] and even mental health through the gut–brain axis [6]. Bacterial communities also influence the whole of human society: they affect the efficiency of wastewater treatment plants [7] and soil microbiomes impact agricultural productivity [8]. Manipulating competitive interactions could thus prove a useful method for managing the composition and function of bacterial communities.

Competition can be described either as exploitation competition, which is indirect and mediated by access to limited nutrients and space, or interference competition, where bacteria directly attack and kill competitors [9]. Bacteria have evolved a wide variety of molecular systems for use in antagonism – weapons [9]. The molecular biochemistry of bacterial weapons has been extensively studied, but many questions remain regarding the ecology and evolution of bacterial antagonism [9]. Central to understanding the ecology of weapon use is regulation: under what biotic and abiotic conditions is it best to use weapons, and what kind of weapon? When considering the evolution of weapon use, the fitness costs of weapon expression are useful for explaining limits on the use and prevalence of different weapons. Non-weapon producers are expected to have an advantage due to an inherent cost in the use of weapons [10–17], and resistance to attacks is also expected to incur a cost [18, 19]. In theoretical work and models, this is generally described as a growth-rate trade-off: bacteria can either invest finite resources to produce more cells or produce weapons (or resist their effects) [12, 20–30]. How such trade-offs manifest at the molecular level within cells is generally not addressed. Understanding the physiological constraints faced by bacterial cells will allow for a clear characterization of the trade-offs incurred by producing or resisting attacks. In this perspective, I will describe recent work on how proteome allocation constraints determine how bacteria manage competing demands on their limited cellular resources and how these insights can be used to better understand interbacterial antagonism.

## Understanding bacterial physiology through proteome allocation constraints

Bacteria produce proteins to perform all necessary cellular functions, including the further production of new proteins. This task is carried out by ribosomes, which consist of about 1/3 protein by mass [31]. Producing a new cell requires the production of ribosomes that will be partitioned into the daughter cell. It could be expected that faster growth could be achieved through faster production of new proteins by adding amino acids to growing peptide chains faster; however, the speed of translation has an upper limit [32]. Therefore, the only way cells can increase their growth rate is to produce more ribosomes, which take up physical space in the cytoplasm [33]. The cytoplasm is densely packed with proteins carrying out their various functions, but the amount of protein per cell volume – the protein concentration – varies little based on growth conditions and even between different kinds of bacteria [33]. Multi-step metabolic pathways function more efficiently with higher protein concentrations, up to a point, but there is a maximum protein concentration which, when exceeded, results in decreased activity for all cellular functions [34, 35]. Inside the bacterial cell, it is the concentration that determines the activity of a protein, not its absolute amount. This brings forth the fundamental constraint of proteome allocation: in order *to produce more ribosomes* to grow faster, a bacterial cell *must make less of some other protein*(s) to keep the protein concentration within acceptable bounds [33]. All cellular proteins thus incur an opportunity cost: the cost of making one protein is that a different protein is not produced. Producing non-ribosomal proteins thus necessarily incurs an opportunity cost of not making more ribosomes and, therefore, possibly limiting the cell’s growth rate. However, a cell cannot reproduce solely using ribosomal proteins: it must also take up nutrients from the environment to satisfy its needs for energy and precursor building blocks (catabolism) and build all the components of a new cell (anabolism). The proportions of proteins involved in these functions are regulated in tight concert with the proportion of proteins used for protein synthesis and are strongly influenced by environmental conditions [33].

**Fig. 1.**
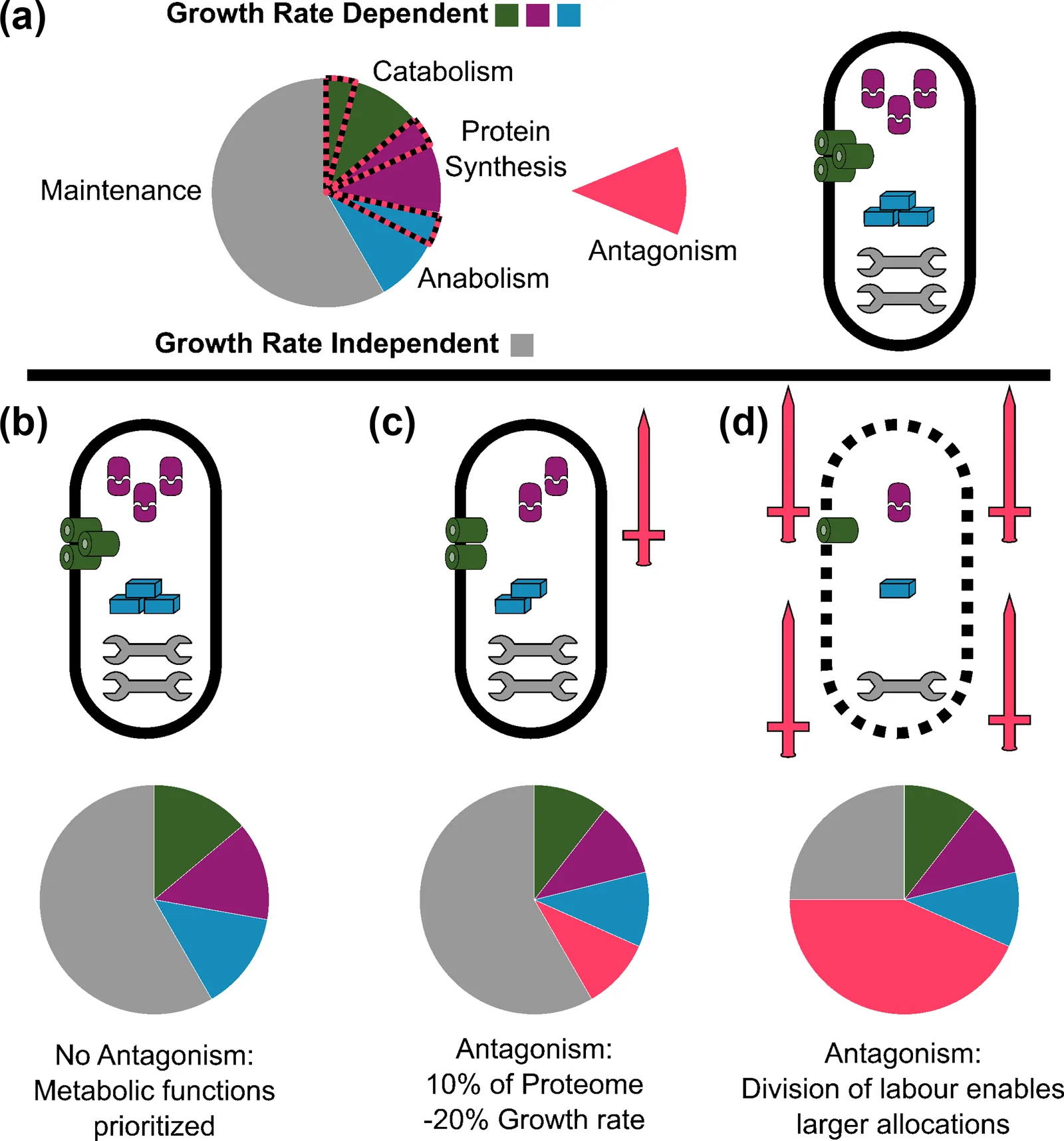
Allocation of proteome resources towards antagonism comes at the cost of other functions. All bacterial proteins from the total proteome can be grouped together based on function into broad ‘sectors’. Some sectors can be categorized as growth-rate dependent, as their relative size shifts depending on the cell’s growth rate, which includes the functions of catabolism, including transporting and breaking down carbon sources; protein synthesis, including ribosomal proteins, elongation and initiation factors, and tRNA synthetases; and anabolism, including all proteins involved in building new cellular components. Growth-rate-independent sectors do not change based on the growth rate and include all other activities needed to keep a cell functioning (here deemed ‘maintenance’). For a cell to produce proteins involved in antagonism, it must make a trade-off in producing fewer proteins from the growth-rate-dependent sectors (**a**). Not investing in antagonism thus allows a cell to prioritize metabolic functions and maximize its growth rate (**b**). By producing antagonistic proteins, a cell’s growth rate must decrease, and it will decrease by approximately double the proportion of the total proteome spent (**c**). In a population of cells where a sub-population differentiates to specialize in the production of proteins involved in antagonism, these cells could bypass the normal constraints of proteome allocation and dedicate most of their proteome towards antagonism, even diminishing essential maintenance functions (**d**).

Gene regulation can be construed as a mechanism cells use to optimize investment of their finite proteome resources. For heterotrophs, proteins involved in the catabolism of carbon sources and anabolism for building cellular components are major constituents of the proteome [33]. Understanding proteome allocation allows for two longstanding, disparate observations about the physiology of *Escherichia coli* to be explained. First, different carbon sources have long been known to enable different growth rates, with glucose yielding a high growth rate while suppressing the expression of other catabolic pathways, known as carbon catabolite repression [36]. Second, the highest growth rates attainable at high concentrations of glucose are achieved not by using the high-ATP-yielding aerobic (Emden–Meyerhof–Parnas) glycolytic pathway, but counterintuitively, the lower-yield fermentative pathway [37]. Both observations have the same explanation: glucose, specifically when assimilated by fermentation, requires a lower investment in catabolic proteins, allowing higher investment in ribosomal proteins and, therefore, higher growth rates [33]. Lower concentrations of glucose or other carbon sources require diversion of resources towards catabolic proteins, which, even if more efficient in their ATP yield, reduce the investment in ribosomal proteins and, therefore, lower the growth rate [37, 38]. Investment in antagonistic proteins must also incur an opportunity cost on the proteome (Fig. 1). The trade-off between growth rate and antagonism is thus a question of how much of the finite proteome is allocated towards antagonistic proteins. As bacterial metabolic strategies are expected to evolve towards proteome efficiency [39], so should their antagonistic competitive strategies. Knowing how proteome allocation constraints exist within the cell, we can develop a new perspective on the fitness costs and trade-offs incurred by bacteria engaging in antagonism and how preparing for and responding to attacks also incur proteomic costs.

For a more in-depth treatment of proteome constraints, in particular the relationship between protein synthesis, anabolism and catabolism and how these are connected to carbon catabolite repression, see the recent review [33]. For a view on how proteome constraints influence cooperative metabolism between community members, see [39]. Here, I present a perspective focused on competitive interactions between bacteria.

## What are the costs of weapon use when considering proteome allocation constraints?

The magnitude of the opportunity cost of producing antagonistic proteins determines the trade-off between attacks and growth rate. In *E. coli*, the trade-off between growth rate and unneeded proteins has been quantified as approximately linear: the relative change in growth rate is ~2× the protein’s concentration (e.g. a bacterium that dedicates 1% of its proteome to antagonistic proteins will grow 2% slower) [38]. Any bacterium that regulates its ribosome levels using guanosine tetraphosphate, like *E. coli*, should thus incur similar costs, as this global regulation strategy pairs ribosome levels with amino acid levels [40]. Theoretical studies that attribute growth rate penalties in the range of 1–20% [22, 24, 26, 27] are thus indicate that these bacteria are dedicating 0.5–10% of their proteome towards the production of antagonistic proteins. For reference, when growing at its maximal rate, *E. coli* allocates ~90% of its variable proteome (~60% of the total proteome is dedicated to functions that do not shift with growth rate) to anabolism/catabolism/ribosomal proteins [41]. At lower growth rates close to 100% of the variable proteome is dedicated to these functions, so 10% of the total proteome being allocated towards antagonism is a very high proportion. Recognizing this may help explain why finding a cost for the type VI secretion system (T6SS) has been difficult and has yielded conflicting results, discussed below. Mapping proteome costs to the production of diffusible interbacterial weapons is more difficult, as many bacteriocins are secreted through self-lysis [42]. How do proteome allocation constraints impact this strategy? Here, individuals in a population may dedicate high proportions of their proteomes to antagonistic protein production, sacrificing their fitness completely for the benefit of their clonemates. This sociobiological phenomenon has been explained in terms of kin selection, where the costly behaviour of an individual is outweighed by its benefit to related individuals [43]. For these kinds of weapons, a population growth-rate penalty may accurately capture the cost of weapon production; however, it has also been observed that cells that shift to terminal programmes of colicin production would have died anyway [44], so there may not even be a population growth-rate cost. This kind of weapon deployment seems to have evolved to allow these cells to use their last moments – and all their proteomic resources – to assist their clonemates. In small-molecule antibiotic-producing actinomycetes, the division of labour where only a subset of cells produce antibiotics has also been observed [45]. While kin selection provides an evolutionary explanation, a shift in investment of proteomic resources provides a mechanistic explanation for why antibiotic production works better with singular cells committing to significant antibiotic production rather than all cells in a group trying to balance antibiotic production and continued growth. Dividing functions between cells allows the normal constraints of proteome allocation to be bypassed, as dedicated antagonistic cells are relieved of the need to optimize their proteomes towards growth and division. Understanding why certain weapon systems have evolved to be deployed this way while others evolved within the proteome allocation constraints of individual cells may lead to a better understanding of interbacterial antagonism.

## Re-examining costs of the T6SS in consideration of proteome allocation constraints

**Fig. 2.**
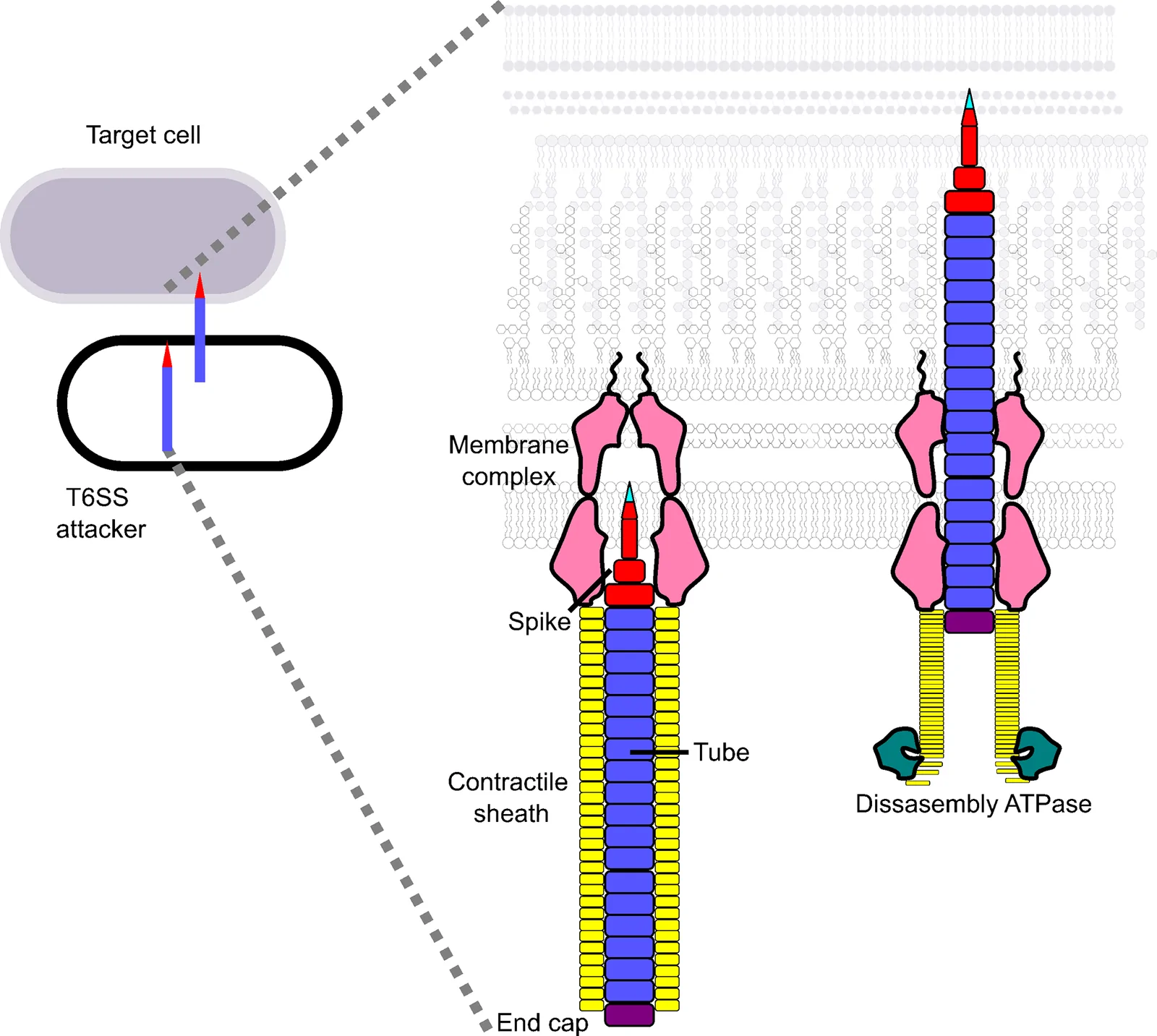
Schematic representation of the T6SS. The T6SS is commonly used as an interbacterial weapon, as attacker cells forcibly inject effector proteins into target cells. It is first assembled inside the producing cell, where the tube is anchored to the membrane complex and surrounded by a contractile sheath. Effector proteins are loaded onto the spike complex and into the tube. Upon contraction, the sheath is propelled out of the attacker cell and pierces through the outer membrane of target cells. Through this process, the effector proteins, which have a wide range of cellular targets and activities [73], are deposited into the target cell. The contractile sheath is then recycled by dedicated ATPases for use in a subsequent firing cycle.

The T6SS is a dramatic example of a complex – and thus presumed costly – bacterial weapon (Fig. 2). It is a contractile nanomachine that forcibly injects effector proteins into adjacent cells. The T6SS is made from >12 different proteins, most of which are repeated as subunits, with a total size in the MDa range [46, 47]. Many proteins are lost during firing events, but the contracted sheath is recycled by an ATPase. Given these features, the use of the T6SS is expected to incur a substantial cost to the cell. These costs have been investigated either in terms of ATP use, creating a metabolic burden on the cell, or more generally as a growth-rate or fitness cost, where a genotype using a T6SS is expected to be outcompeted by a non-producing, non-susceptible genotype. Despite its large size and complexity, several studies have found that the costs are small or indistinguishable from zero [14–16]. To my knowledge, the costs of T6SS production have not been considered in terms of proteome allocation opportunity cost, so here we will examine whether proteome allocation constraints can help explain these results.

The T6SS can be regulated at many different levels, which could influence how costly it is for a producing cell. Taillefer and colleagues used mutant strains of enteroaggregative *E. coli* to interrupt its T6SS at the levels of expression and synthesis of the constitutive proteins, assembly of the sheath, contraction and disassembly [14]. Based on our understanding of proteome allocation constraints, it could be expected that only disruption of gene expression and protein synthesis would allow diversion of the resources used for the T6SS towards other functions. However, none of the mutants had higher growth rates than the wild-type, even in the presence of a variety of environmental stressors, nor could they outcompete the wild-type in direct competition (the mutants were not susceptible to attack). The authors concluded that the number of polypeptides used to construct a T6SS (~2.3×10^3^ proteins) and ATP costs (10^6^–10^7^) were negligible compared to the size of the cellular proteome (3–10×10^6^ proteins) and ATP pool (3×10^12^). A similar result was observed in *Vibrio cholerae*; however, the cost was measured by co-culturing a strain constitutively expressing its T6SS with a non-producer for ~100 generations [15]. Here, Zhang and colleagues concluded that, in the most likely cases, the metabolic cost was so low that, in a population where beneficial mutations occur in different lineages (clonal interference), competition between lineages prevents the cost of T6SSs from incurring a noticeable penalty [15]. They concluded that T6SS expression would have to incur a growth-rate cost of 0.5% (i.e. 1% of the total proteome) for it to be noticeable under certain circumstances [15]. Both groups considered the cost of the T6SS in terms of metabolic cost rather than the opportunity cost of producing the T6SS instead of some other cellular function. As observed in *E. coli,* lower-ATP-yielding pathways can be preferred if their proteome cost is lower [37], so measuring the costs of the T6SS in terms of ATP consumption likely does not capture its true cost.

By considering proteome allocation costs, both results indicate that the recovered proteome allocation was too small to be measured as an increase in growth rate. For *E. coli*, the proportion of the proteome spent on a single T6SS would range between 0.023 and 0.077% [14], which might correspond to a growth-rate cost of 0.046–0.154%, somewhat higher than what was measured for constitutive expression of the T6SS in *V. cholerae* (0.025%) [15]. If the higher estimate of the cost of a single T6SS is taken, and cells produced four T6SSs, then based on Zhang and colleagues’ work [15], the fitness-cost threshold of 0.5% would be breached, which may allow selection against producers to be detected after a few hundred generations. By considering how much of the proteome is spent on T6SSs, this small investment likely does not incur a detectable growth-rate cost in most scenarios. When considering the benefit, which has been measured as providing advantages over competitors of 10^5^-fold [20], it is clear that this small growth-rate cost is well worth the proteome allocation spent. Theoretical studies should consider this when attributing penalties for contact weapon production. Still, costs of the T6SS have been measured under more realistic conditions, which will now be discussed.

*Vibrio fischeri* uses its T6SS to kill competitors while colonizing its squid host [48]. Septer and colleagues investigated the costs of producing and firing T6SSs, predicting that ATP-equivalent costs were a higher proportion of the ATP budget in stationary phase compared to exponential growth [17]. Matching these predictions, they also found that a non-expressing mutant grew at an indistinguishable rate but reached a higher total density than a T6SS-producing strain [17]. They posit that the regulatory mutant was ‘able to divert additional energy into biomass’; however, it may be that this strain re-allocated its proteome towards ribosomal proteins, increasing its growth rate. *Bacteroides fragilis* also uses its T6SS for competition while colonizing its niche in the mammalian gut [49], but strains with inactivating mutations are regularly isolated, indicating that in natural conditions T6SS production incurs a cost [16]. Robitaille and colleagues thus investigated the costs of T6SS use between producing and non-producing strains and found that costs manifested only in the natural conditions of the mouse gut microbiome but not in laboratory culture [16]. During these host colonization conditions, the T6SS-producing strain had an advantage as long as there was a sensitive strain to outcompete; however, it was outcompeted by the resistant, non-producing strain in the absence of the sensitive strain. This result indicates that it may not be the proteomic changes in the attacker strain that caused it to be outcompeted, but changes in its competitors.

## Implications of proteome allocation constraints on cells being attacked

Bacteria have engaged in antagonism throughout their evolutionary history, so have also evolved to withstand attacks [50]. Indeed, clinically important antibiotic resistance determinants have existed for far longer than the large-scale use of antibiotics [51]. While some bacterial defence mechanisms provide general protection to threats such as eukaryotic and phage predators [50], others are specific to interbacterial attacks. *Bacteroides* use a recombinase-based system to acquire large arrays of immunity proteins to protect themselves from T6SS attacks [52], while *Pseudomonas aeruginosa* uses a dedicated set of proteins to detect incoming attacks to optimally deploy its own T6SS counterattacks [53–55]. Expression of these defensive genes is often under the control of competition sensors, which detect signals such as kin-cell lysis [56, 57]. Production of the proteins for these systems must also come at the cost of making fewer proteins involved in growth, which likely explains why their expression is tied to situations when they are expected to be beneficial. Proteome allocation trade-offs also show how attacks do not need to be lethal to greatly decrease growth rate. Sub-inhibitory concentrations of antibiotics substantially change gene expression [58], and inhibition of ribosomes by chloramphenicol leads to much greater investment in ribosomal proteins [38]. This may help explain the vast diversity of toxin targets [59]: any attack that forces a shift in proteomic resource allocation will have double the effect on the growth rate, so deploying a weapon that is highly likely to have some effect is more useful than deploying a weapon that prioritizes killing the target, which may be more difficult in the face of immunity and other protective mechanisms.

## How might proteome allocation constraints have influenced the evolution of weapons?

If proteome allocation constraints explain the costs of investing in antagonism, then these same constraints may help explain why evolution has produced the wide variety of different weapon systems observed in bacteria [9]. The function of a bacterial weapon system is to deliver a toxic molecule onto or into a competitor’s cell. These toxic molecules can range in composition and size from small-molecule antibiotics to heavily modified peptides to multiprotein complexes [9]. In addition to direct injection mechanisms like the T6SS, bacteria have evolved a range of secretion mechanisms to export toxins, which enter target cells either through active or passive transport [9]. Proteome allocation constraints may thus manifest in the form of the toxic effectors, the methods by which they are secreted, and how they are regulated. Regulation itself can be described as a mechanism to reduce the proteomic costs of producing weapons by only expressing antagonism genes under certain conditions. Only doing so when other bacteria are present, so-called competition sensing, is theoretically expected to be the most effective strategy [26], although quorum-sensing [60], constitutive expression [61], and as discussed earlier, division of labour [45], are also known. As regulation was discussed above, here we will discuss how proteome allocation constraints may affect the form and secretion pathways of some bacterial weapons.

Bacteria use a variety of short-range weapons other than the T6SS [9], which could be influenced by proteome allocation constraints. The type IV secretion system (T4SS) can be used to inject toxic effector proteins into competitor’s cells [61]. Recently, an interesting observation was described where many lineages of *Xanthomonas translucens* shifted from using a T4SS to a T6SS for close-range combat [62]. The T4SS is ancestral in this species, but strains that acquired a T6SS with anti-bacterial effectors by horizontal gene transfer also gained mutations, which caused them to stop T4SS expression [62]. This indicates that there was a selective advantage to ceasing production of the T4SS, which could reflect higher proteomic costs than the T6SS. The T4SS requires formation of a pilus bridge between the attacker and target cells, which, in addition to a secretion complex, may be more inefficient than the T6SS. Contact-dependent inhibition (CDI) is also a short-range weapon – a type V secretion system – which consists of just two proteins: the outer membrane auto transporter and the toxin-bearing filament [63]. *P. aeruginosa* strains regularly encode multiple CDI systems, in addition to their multiple T6SSs [64], without mutation leading to degradation. While CDI appears to require fewer proteomic resources at first glance, each filament can be over 5,500 aa long (e.g. PAO1 CDI2) [65] and only allows delivery of a single toxin effector compared to the multiple effectors delivered simultaneously by the T6SS [66], though it is extracellular so would not incur an opportunity cost for protein concentration. Comparison of proteomic cost differences between these different weapon systems may thus require careful accounting of the number of toxin units deliverable per unit of proteome investment, and degree of effect on the target cell per toxin. Still, it is possible that CDI represents a lower proteome cost weapon for use in conditions where the higher cost weapons are unsustainable.

**Fig. 3.**
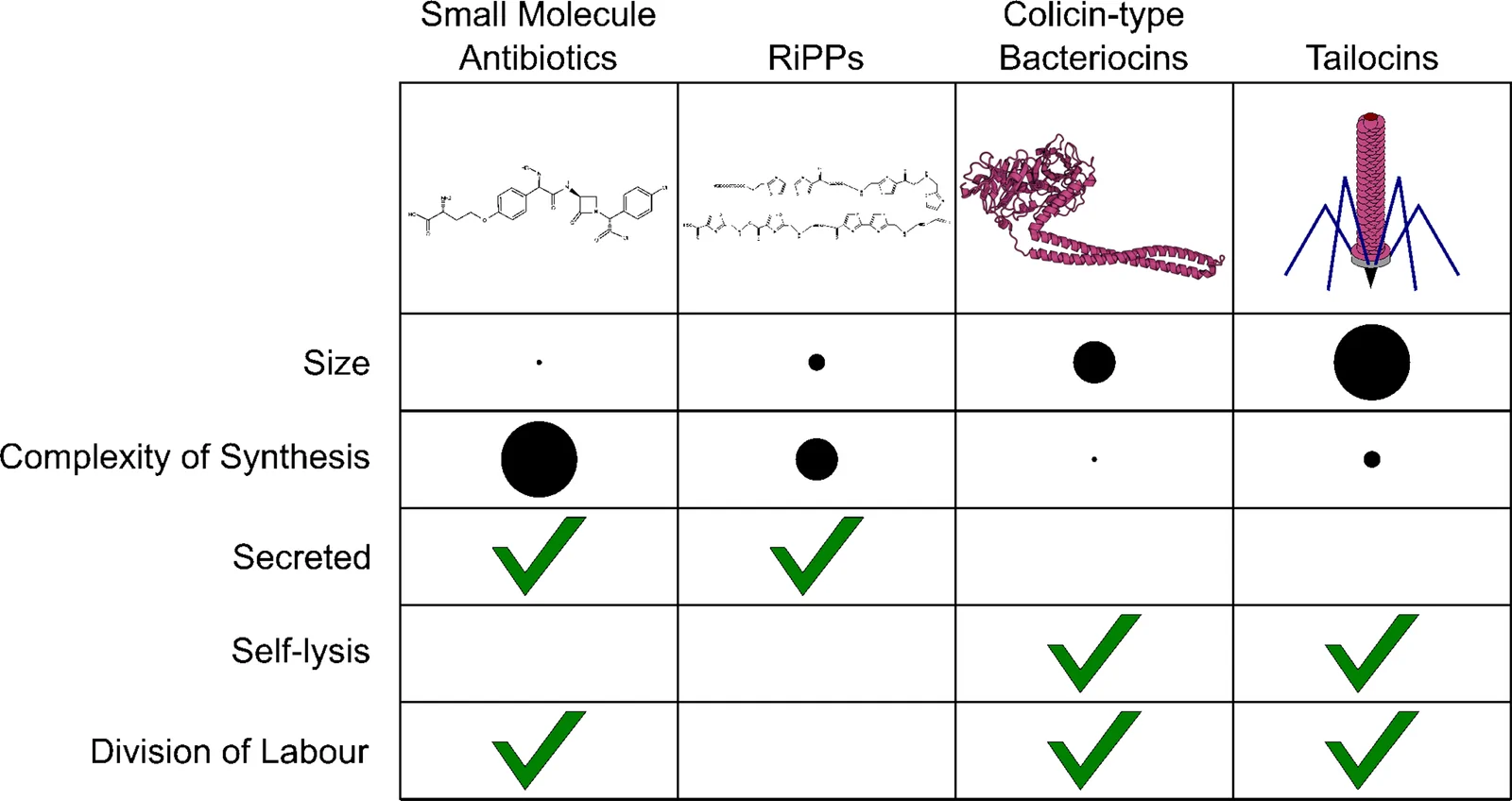
Long-range diffusible bacterial weapons differ across multiple properties. Bacteria produce and secrete a range of molecules for attacking competitors at a distance. These include small-molecule antibiotics such as nocardicin A [74], RiPPs such as microcin B17 [75], ribosomally synthesized multi-domain protein bacteriocins, which are similar to *E. coli’s* colicins such as colicin E3 [76] and phage tail-derived multiprotein complex tailocins such as pyocin R2 [77]. These weapons vary substantially in their size and the number/complexity of enzymes involved in their production, i.e. their complexity of synthesis. Small-molecule antibiotics are synthesized by NRPs, while RiPPs use a genomically encoded scaffold peptide that is modified by several enzymes after translation. Colicin-type bacteriocins are encoded by a single gene, whereas tailocins are built from multiple structural proteins that oligomerize. Small-molecule antibiotics and RiPPs are secreted by the producing cells, whereas colicin-type bacteriocins and tailocins are released into the environment by self-lysis of the producing cell. Since only a small subset of the population engages in this altruistic behaviour, it entails division of labour at the population level. Similarly, actinomycetes producing small-molecule antibiotics are known to differentiate a portion of their cells into non-dividing, antibiotic-producing cells [45]. This kind of behaviour has not been described for RiPPs. Note that the properties described here are generalizations and may not hold strictly true in all cases.

Bacteria attack and kill competitors at a distance by producing and secreting diffusible small-molecule antibiotics and a variety of peptides and proteins that differ in several properties [9] (Fig. 3). Many small-molecule antibiotics are synthesized by non-ribosomal peptide synthetases (NRPS) [67] that can produce complex chemical structures such as lactam rings and are secreted by dedicated transporters. Ribosomally synthesized, post-translationally modified peptides (RiPPs) also contain complex chemical moieties but are built from a peptide scaffold, then altered by modification enzymes and also have cognate secretion proteins [68]. Many antibacterial molecules produced by such enzymes have been called ‘bacteriocins’, though this term has also been applied to the unmodified, ribosomally synthesized single-protein colicins (and similar proteins), as well as phage-tail-derived multi-protein complexes that kill through contraction of the phage tail (tailocins) [9]. Both colicin-type bacteriocins and tailocins are ‘secreted’ through sacrificial self-lysis of the producing cell, invoking kin selection and division of labour [42, 69, 70]. Proteome allocation constraints have likely influenced both the form and secretion mechanisms of these weapons. Some small-molecule antibiotics produced by actinomycetes are known to be produced through division of labour [45], possibly due to the large proteome investment needed to make the biosynthetic NRPSs. Conversely, the RiPPs like lantibiotics and microcins are not known to be produced through division of labour [71] and may be produced more uniformly in a population, possibly due to their production by just a few biosynthetic enzymes incurring a low proteome cost. The RiPPs can also be distinguished from the much larger colicin-like bacteriocins, which only require a single gene to be transcribed and translated to produce a toxic protein but are released through self-lysis, even though bacteria could secrete proteins of this size [72]. It is unclear whether lytic secretion is contingent or has been selected for, but it is clear that this strategy involves division of labour, allowing producing cells to bypass normal proteome allocation constraints. Further consideration of these constraints may yield insights into why diffusible bacterial weapons have taken the wide variety of forms that have been observed.

## Conclusion

Bacterial physiology operates within the constraints of a finite protein budget and maximum translation speed. These constraints influence all functions of the cell, as allocating proteome resources towards one function necessitates allocating fewer resources to other functions. This is especially important with regard to growth and division, as decreasing investment in these functions decreases the growth rate of the cell. This framework can thus be used to better understand how bacteria engaging in antagonism incur costs: investing in attacks requires decreasing investment in other functions. Proteome allocation constraints can also help us understand why bacterial weapons take the forms they do: peptide bacteriocins can be produced by assembly lines of proteins, allowing a small investment by all cells in a population to produce enough toxin. However, it may still be more efficient to produce small-molecule antibiotics by having some cells within a population differentiate into dedicated factories, where growth is greatly diminished in exchange for a larger proportion of the proteome being dedicated to antibiotic production. This leads to sociobiological implications as the producing cell must have its benefits accrued by closely related siblings. This division of labour can also allow for the production of protein bacteriocins with extremely high potencies. Differences in the proteomic costs of weapon production may also manifest in the choice of weapon used: the T6SS may be more efficient than the T4SS for directly injecting effectors into competitors, so using both may be inefficient, whereas CDI is proteome-efficient enough to allow use of both CDI and the T6SS. These are just a small beginning of the possibilities of using proteome allocation constraints to better understand interbacterial antagonism. Applying this knowledge should be especially helpful when designing studies to determine the costs of weapon production.

## Abbreviations

CDI: contact-dependent inhibition
NRPS: non-ribosomal peptide synthetases
RiPP: ribosomally synthesized, post-translationally modified peptide
T4SS: type IV secretion system
T6SS: type VI secretion system
